# Thymoma associated with hypogammaglobulinaemia and pure red cell aplasia

**DOI:** 10.3332/ecancer.2013.364

**Published:** 2013-10-17

**Authors:** Juan Briones, Mirentxu Iruretagoyena, Héctor Galindo, Claudia Ortega, Pablo Zoroquiain, José Valbuena, Francisco Acevedo, Mauricio Ocqueteau, Cesar Sánchez

**Affiliations:** 1 Department of Hematology-Oncology, School of Medicine, Pontifical Catholic University of Chile, Chile 8330024; 2 Department of Clinical and Rheumatologic Immunology, School of Medicine, Pontifical Catholic University of Chile, Chile 8330024; 3 Department of Radiology, School of Medicine, Pontifical Catholic University of Chile, Chile 8330024; 4 Department of Pathological Anatomy, School of Medicine, Pontifical Catholic University of Chile, Chile 8330024

**Keywords:** thymoma, pure red cell aplasia, hypogammaglobulinaemia

## Abstract

Thymomas are neoplasias that begin in the thymus and develop in the anterior mediastinum. They are commonly associated with a variety of systemic and autoimmune disorders, such as pure red cell aplasia, hypogammaglobulinaemia, pancytopaenia, collagen diseases, and, most commonly, myasthenia gravis. The presence of inter-current infections, especially diarrhoea and pneumonia, in the presence of lymphocyte B depletion and hypogammaglobulinaemia is known as Good’s syndrome and may affect up to 5% of patients with thymoma. While anaemia is present in 50%–86% of patients with Good’s syndrome, only 41.9% of cases present pure red cell aplasia. Concomitance of these two conditions has only been rarely studied.

We report on the case of a 55-year-old man diagnosed with advanced thymoma, who, during the progression of his disease, developed signs and symptoms suggesting Good’s syndrome and pure red cell aplasia. We also performed a brief review of the literature concerning this association, its clinical characteristics, and treatment.

## Introduction

Thymomas are not common neoplasias, with an incidence of 0.15 cases per 100,000 people per year. They represent 20%–30% of mediastinal tumours in adults [1, 2]. Patients usually present with symptoms secondary to local compression, such as respiratory symptoms or superior vena cava syndrome. On other occasions, they may appear with a paraneoplastic syndrome [2], including myasthenia gravis, pure red cell aplasia, connective tissue disorders, and hypogammaglobulinaemia/Good’s syndrome [1, 2]. Good’s syndrome affects at least 5% of patients with thymomas [3]. The principal immunological abnormalities described for this syndrome include hypogammaglobulinaemia, B-cell lymphopaenia, CD4/CD8 ratio inversion, low T CD4 lymphocyte counts, and alteration of the mitogenic T cell response [4]. Pure red cell aplasia is described in less than 10% of patients with thymoma and is characterised by an erythropoiesis insufficiency, with granulopoiesis and megakariopoiesis conservation [11]. Pure red cell aplasia may be the initial finding that leads to the diagnosis of thymoma or may develop after diagnosis. 

We report the case of a 55-year-old man, diagnosed with advanced thymoma, who developed hypogammaglobulinaemia/Good’s syndrome and pure red cell aplasia as his condition evolved.

## Clinical case

**Figure 1.**
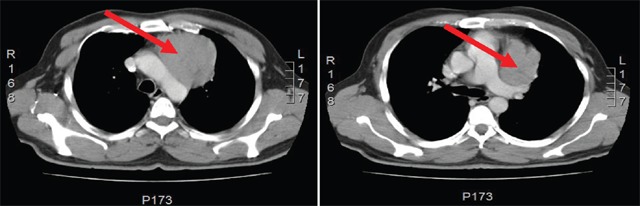
Computed Tomography of the chest shows the presence of an anterior mediastinal solid mass (red arrow) that has a maximum diameter of 7.5 cm. This tumor has a fine line of separation from adjacent vascular structure and back side of the sternum and rib cartilage.

**Figure 2.**
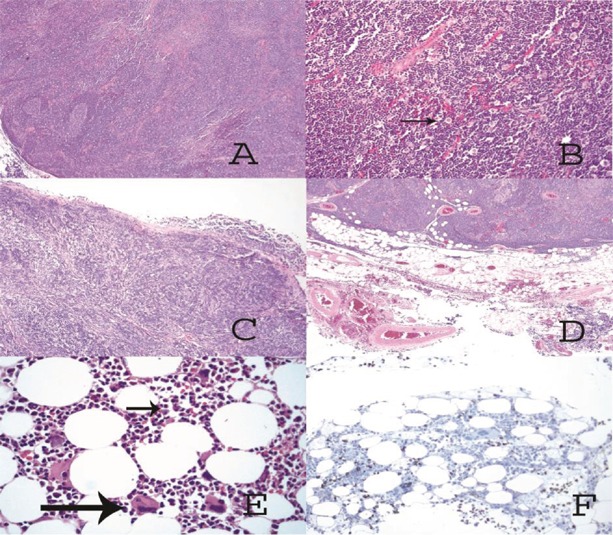
(A) Solid tumor made up of lymphocytes with germinal center formation. (B) At higher magnification, (arrow) large cells of clear eosinophilic cytoplasm and round nuclei (epithelial cells) are also observed. (C) The tumor has invaded the pleura, passing through it. (D) The tumor has invaded the mediastinal adipose tissue. (E) In the hypocellular bone marrow only megakaryocytes (big arrow) and myeloid elements are observed (short arrow). (F) Immunohistochemical staining against glycophorin C is only positive on red cells; no erythroid precursor-like cells are observed.

The case involved a 53-year-old male patient, with a history of type 2 diabetes mellitus 2 and dyslipidaemia. He consulted his doctor for a month-long condition of dry cough associated with a loss of weight amounting to 10 kg. A chest x-ray showed a rise of the left hemidiaphragm. A computed tomography (CT) scan of the thorax showed an anterior mediastinal tumour, with no evidence of spreading to adjacent tissue (Figure 1). A biopsy of the mediastinal tumour, guided by the CT, was performed, confirming the diagnosis of thymoma, which was treated by thymectomy that left residual macroscopic disease. A biopsy of the surgical piece showed type B1 thymoma, according to the World Health Organization (WHO) classification, with infiltration into the sub-pericardial connective tissue and mediastinal adipose tissue, associated with pulmonary implants (Figure 2AD) Masaoka stage III. Positron emission tomography-computed tomography (PET-CT) control after three months showed no evidence of local or systemic spread of the thymoma, and so it was decided to maintain clinical controls. During the followup period, the patient presented with two episodes of pneumonia.

**Figure 3.**
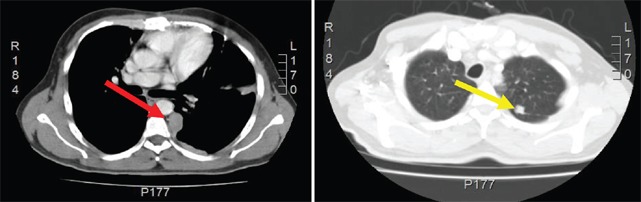
Computed Tomography of the chest shows left pleural nodular thickening predominantly in costophrenic recesses, identifying large tumors at this level (red arrow). Multiple left juxta-cisural nodules up to 13 mm (yellow arrow).

A year after surgery, the patient developed exercise-induced dyspnoea, associated with a progressive deterioration in functional capacity. The chest CT (Figure 3) showed left nodular pleural thickening, associated with pulmonary ipsilateral and adenopathic mediastinal nodules, compatible with a diagnosis of tumour recurrence. Due to symptomatic thymoma progression, palliative cyclophosphamide, doxorubicin, cisplatin, and prednisone combination chemotherapy (CAPPr) was initiated.

Prior to the beginning of treatment, the patient developed the signs and symptoms of pneumonia and responded favourably to treatment with antibiotics. After the first and second cycles with CAPPr, the patient had to be hospitalised with febrile neutropaenia; both times, it was satisfactorily treated with antibiotics and colony-stimulating factors. After the third cycle with CAPPr, the patient was once again hospitalised, due to symptoms of abdominal pain and abundant diarrhoea (30 episodes per day), containing mucous and blood. A general laboratory analysis did not detect neutropaenia (Table 1). The microbiological study was negative. The study was completed with a colonoscopy that showed an isolated blind ulcer, the biopsy of which showed the presence of lamina propria micro-haemorrhaging. Suspecting acquired hypogammaglobulinaemia, an immunoglobulin count (Table 2) and lymphocyte subpopulation analysis (Table 3) were requested, which confirmed the diagnosis of Good’s syndrome. As a result, the patient was given an intravenous immunoglobulin supplement. He was discharged in good condition to continue outpatient management.

Three months later, the patient was again hospitalised with the signs and symptoms of anaemic syndrome. The complete blood count (CBC) showed severe arregenerative anaemia (Table 1). He was given a transfusion of red blood cells, and a bone marrow biopsy was performed, showing typical findings consistent with pure red cell aplasia. (Figure 2E,F). Evaluation revealed radiological progression of the disease, so chemotherapy with second-line drugs paclitaxel and carboplatin was given. Since his release, he has not returned to the clinic.

### Discussion

The relationship between thymoma and hypogammaglobulinaemia was initially described by Dr Robert Good in 1954 [4]. At the present time, there are various definitions for Good’s syndrome. Some authors define it as a subtype of common variable immunodeficiency (CVID); however, the depletion of peripheral B lymphocytes associated with Good’s syndrome is not characteristic of CVID, which is typically associated with an alteration in B lymphocyte maturation [5, 6]. Others define it as hypogammaglobulinaemia associated with thymoma, which is consistent with the case originally described by Dr Good [5].

As previously mentioned, patients with Good’s syndrome have, in the presence of thymoma, B cells missing or diminished in peripheral blood, hypogammaglobulinaemia and cellular immunity defects [2, 7]. Immunodeficiency is combined, affecting the humoral response as well as the cellular, predisposing patients to upper and lower respiratory infections, similar to CVID and also to opportunistic infections observed in HIV-positive patients [2, 8, 9]. Good’s syndrome most often affects patients between 40 and 70 years of age [2], affecting both sexes equally and occurring in less than 5% of patients with thymoma [4]. As to prognosis, 70% of Good’s syndrome patients will be alive after five years compared with almost 100% survival for patients with agammaglobulinaemia related to the X chromosome and with CVID [4, 16].

According to a systematic review of the literature, diagnosis of thymoma precedes documentation of hypogammaglobulinaemia, infection, or diarrhoea in 42% of patients. In this study, thymoma was observed after diagnosis of hypogammaglobulinaemia or infection in 19.7% of cases. Around 37.9% of the population was diagnosed simultaneously [8].

The pathogenesis of Good’s syndrome is unknown. However, at least three hypotheses have been put forth: (1) cytokines: the first hypothesis arose from murine models, which demonstrated that cytokines such as limitin, an interferon-type cytokine produced by a stromal cell line in the bone marrow, is capable of influencing the growth and differentiation of B cell precursors, causing detention and alteration of cellular maturation [5, 10]; (2) many patients with Good’s syndrome are affected by opportunistic infections associated with cellular immunity defects, arising from the loss of memory or ‘naïve’ T CD4 cells [4, 5]; and (3) autoimmunity. Studies on paraneoplastic syndromes such as pure red cell aplasia with thymoma show that auto-antibodies or auto-reactive T lymphocytes could directly or indirectly inhibit erythropoiesis, so that the loss of B cell function could be secondary to the immune destruction [5].

The most common infection in these patients is recurrent pneumonia, secondary to encapsulated organisms (humoral response). They also develop opportunistic infections such as retinitis or colitis caused by cytomegalovirus, mucocutaneous infection caused by candida, pneumonia from *Pneumocystis jiroveci*, among others (cellular response) [4, 9].

Diarrhoea was reported in almost half of patients with Good’s syndrome. The mechanisms through which hypogammaglobulinaemia and thymoma cause diarrhoea are not very clear. It has been suggested that it could be related to vellositary atrophy, which could be resolved by re-establishment of the immunological state. On the other hand, patients are especially susceptible to gastrointestinal pathogens. Still, often no germ is identified. Among the cases in which it was possible to isolate a bacterial agent, enterobacterias were the most common pathogens. Other pathogens isolated were *Giardia lamblia* and cytomegalovirus [2, 4] 

Treatment with immunoglobulin replacement has been reported in numerous cases of patients with Good’s syndrome, and around 38% show a decrease in the number of infections after treatment [2, 8]. This treatment is also associated with a decreased use of antibiotics and in the number of hospital admissions. Other forms of therapy reported in the literature include immunosuppressant treatment, splenectomy, and plasmapheresis [8, 13].

Autoimmune diseases such as pure red cell aplasia, myasthenia gravis, diabetes mellitus, ulcerative colitis, and haemolytic anaemia may occur in association with Good’s syndrome [2]. According to a systematic review by Kelesidis *et al*, pure red cell aplasia and myasthenia gravis are the autoimmune pathologies most commonly associated with Good’s syndrome, occurring in 34.8% and 15.7% of cases, respectively [8]. This association, as mentioned previously, suggests the potential role of autoantibodies or immunity by cells, in the pathogenesis of hypogammaglobulinaemia [5].

Pure red cell aplasia is often diagnosed soon after the diagnosis of thymoma. In the Mayo Clinic, the majority of patients are diagnosed within a month of being diagnosed with thymoma and the rest between four and 117 months after thymoma resection [11].

According to a review of the existing literature, treatment for pure red cell aplasia associated with thymoma is based on managing the base pathology, supportive transfusion therapy, and immunosuppressant treatment [11, 12, 14, 15]. 

In our case, the patient was diagnosed with Good’s syndrome and pure red cell aplasia two years after being diagnosed with thymoma in relation to a recurring tumour and in the context of inter-current infections associated with anaemic syndrome.

Currently, there are no solid data in the literature in relation to the treatment of patients with Good’s syndrome, pure red cell aplasia, and thymoma. With our patient, we decided to treat the recurrent base pathology with chemotherapy, intravenous gammaglobulin therapy, and transfusion support therapy as required.

### Conclusion

The association of Good’s syndrome with pure red cell aplasia in the context of thymoma has been only scarcely reported in the literature. The presence of inter-current infections associated with severe anaemia with no clear aetiology should lead to a suspicion of this condition.

## Figures and Tables

**Table 1.** General laboratory analysis.

Laboratory	At the end of the third cycle of QMT	Most recent hospitalisation	Reference values
Haemoglobin	10.8	5.6	13.5–17.5 g/dL
Reticulocytes		0	0.5%–1.5%
Ferritin		1062 ng/mL	22–322 ng/mL
Serum iron		363 μg/dL	33–193 μg/dL

**Table 2.** Immunoglobulin counts.

Immunoglobulin counts	Results (mg/dL)	Reference values (mg/dL)
IgG	479	700–1600
IgA	85	70–400
IgM	27	40–230

**Table 3.** Lymphocyte subpopulation.

Absolute lymphocyte subpopulation count	Results	Reference values
Total T lymphocytes	851.7 × mm3	700–2100 × mm3
CD8 T lymphocytes	492.8 × mm3	200–900 × mm3
CD4 T lymphocytes	295.9 × mm3	300–1400 × mm3
B lymphocytes	0 × mm3	100–500 × mm3
NK cells	73.5 × mm3	90–600 × mm3
